# Drug-Resistant Tuberculosis Outcomes in South Kivu Province, Democratic Republic of Congo, During a Humanitarian Crisis (2018–2024)

**DOI:** 10.1093/ofid/ofag303

**Published:** 2026-05-14

**Authors:** Eric Mulume Musafiri, Barhwamire Théophile Kabesha, Freddy Birembano, Rosette Nyota, Maryska Chimanuka Mapendo, Jean Paul Chirambiza, Trésor Kashinde Mosomo, Edmond Ntabe Namegabe

**Affiliations:** Drug Resistance Unit, National Tuberculosis Program Democratic Republic of the Congo (DRC), Bukavu, DRC; Department of Medicine, Université Officielle de Bukavu, Bukavu, DRC; Department of Public Health, Institut Supérieur de Techniques Médicales de Kaziba, Kaziba, DRC; Department of Medicine, Université Officielle de Bukavu, Bukavu, DRC; Department of Public Health, Institut Supérieur de Techniques Médicales de Kaziba, Kaziba, DRC; Faculté de Médecine, Université Officielle de Bukavu, Bukavu, DRC; Drug Resistance Unit, National Tuberculosis Program Democratic Republic of the Congo (DRC), Bukavu, DRC; Drug Resistance Unit, National Tuberculosis Program Democratic Republic of the Congo (DRC), Bukavu, DRC; Department of Internal Medicine, Hôpital Général de Référence de Miti-Murhesa, Sud Kivu, DRC; Expertise France, Project RESOH-Lab, Bureau de Bukavu, Bukavu, DRC; Public Health School of Goma, University of Goma, Goma, DRC; Department of Medicine, Université Libre des Pays de Grands Lacs, Goma, DRC

**Keywords:** DR-TB, humanitarian settings, treatment outcomes

## Abstract

**Background:**

Drug-resistant tuberculosis (DR-TB) management in fragile, conflict-affected settings remains challenging. South Kivu Province has faced protracted insecurity and recent outbreaks (COVID-19, mpox). We assessed treatment outcomes and predictors among DR-TB patients managed under programmatic conditions.

**Methods:**

We conducted a retrospective provincial cohort study of all patients registered in electronic Tuberculosis registry with DR-TB in South Kivu from January 2018 to June 2024. Primary outcome was treatment success; unsuccessful outcomes. We summarized characteristics, performed Kaplan–Meier functions for time to outcome, and fitted multivariable logistic regression and Cox proportional hazards models to identify predictors of unsuccessful outcomes.

**Results:**

Among 277 DR-TB patients (median age 37 years; 3–75), overall treatment success was 77.6%, mortality 13.4%, and loss to follow-up (LTFU) 8.6%. HIV status was negative in 79.1% and positive in 9.4%. Bedaquiline-containing regimens were used in 100%. In adjusted analyses, rural residence was protective against unsuccessful outcome (aOR 0.42; 95% CI 0.21–0.82; *P* = .011), whereas retreatment other than relapse was associated with higher odds of unsuccessful outcome (aOR 2.04; 95% CI 1.09–3.81; *P* = .026). Findings were consistent in time-to-event models (rural aHR 0.50; 95% CI 0.29–0.85; *P* = .011; retreatment aHR 1.71; 95% CI 1.03–2.83; *P* = .038).

**Conclusions:**

In a humanitarian crisis context, programmatic all-oral DR-TB care achieved success exceeding the WHO ≥ 75% target. Persistently high mortality and LTFU—concentrated among urban residents and retreatment (non-relapse) cases—highlight the need for targeted urban patient-tracing, strengthened referral/continuity mechanisms, and uninterrupted drug supply. Results support scale-up of short, all-oral regimens with context-specific adherence support in fragile settings.

Tuberculosis (TB) remains a major global health threat and, in 2023, likely regained its position as the leading cause of death from a single infectious agent after 3 years of being surpassed by coronavirus disease (COVID-19) [1]. The burden is increasingly compounded by the emergence of drug-resistant tuberculosis (DR-TB), which undermines global TB control efforts. Drug-resistant tuberculosis encompasses several categories: rifampicin-resistant TB (RR-TB); multidrug-resistant TB (MDR-TB), defined as resistance to both rifampicin and isoniazid; pre-extensively drug-resistant TB (pre-XDR-TB), characterized by MDR-TB with additional resistance to any fluoroquinolone; and extensively drug-resistant TB (XDR-TB), which includes MDR-TB plus resistance to a fluoroquinolone and at least 1 group A drug such as bedaquiline (BDQ) or linezolid (L) [2, 3]. In 2024, the World Health Organization (WHO) ranked MDR/RR-TB among its global priority pathogens, highlighting the urgent need for improved prevention, diagnosis, and treatment strategies [4].

Recent advances in therapeutics, particularly the introduction of fully oral, shorter regimens such as bedaquiline–pretomanid–linezolid (BPaL) and bedaquiline–pretomanid–linezolid–moxifloxacin (BPaLM), have transformed the management of DR-TB. These regimens are more effective, less toxic, and easier to administer than older injectable-based regimens, resulting in improved patient adherence and better outcomes [5]. However, concerns remain regarding optimal patient selection, stewardship of new drugs, and the potential risk of amplifying resistance [6].

Globally, treatment success (cured and treatment completed) for MDR/RR-TB improved to 68% in 2023, yet fewer than half of the estimated 400 000 people who developed MDR/RR-TB initiated treatment that year [1]. Achieving the 2025 and 2030 milestones of the End TB Strategy will require timely diagnosis, universal access to WHO-recommended regimens, and strong continuity of care, which are particularly difficult to sustain in fragile and conflict-affected settings [7].

The Democratic Republic of Congo (DRC) is among the 30 high TB burden countries. In South Kivu Province, programmatic management of DR-TB began in 2012 with the roll-out of GeneXpert diagnostics. An earlier province-wide cohort study (2012–2017) reported a treatment success rate of about 71%, achieved under conditions of substantial donor support [8]. Since then, the province has experienced reduced external funding, disruptions in healthcare delivery linked to the COVID-19 pandemic and, more recently, the mpox outbreak, as well as recurrent insecurity and population displacement [9]. Extending the existing humanitarian crisis, 2025 witnessed a significant escalation in armed violence involving M23 and Wazalendo forces. This resulted in the mass displacement of over 200 000 people and widespread sexual violence, particularly in the Kalehe, Fizi, Mwenga, and Kabare regions [10]. Individuals forced to flee, or those displaced by the conflict, face extreme vulnerability, with mortality rates substantially higher than the general population [11].

These challenges threaten the continuity of care for DR-TB patients, particularly in urban centers and mining zones where mobility is high.

In this study, we report treatment outcomes for DR-TB patients in South Kivu between 2018 and 2024 and identify patient- and program-level factors associated with unsuccessful outcomes.

## METHODS

South Kivu is one of 26 provinces in the eastern DRC, spanning 65 128 km^2^ with an estimated population of 9.2 million in 2025. The province includes 34 health zones, 135 diagnostic and treatment centers for TB (DTCs), and 19 GeneXpert platforms [12].

We conducted a retrospective cohort study of all patients diagnosed with DR-TB between January 2018 and June 2024. Data were extracted from the electronic registry of the Provincial Leprosy and Tuberculosis Coordination, which routinely captures sociodemographic, clinical, and treatment information. This was a census of all eligible DR-TB notifications during the study period; no priori sample size calculation was performed. We included all patients diagnosed with DR-TB, regardless of whether they initiated a short-course all-oral regimen. Patients treated with injectable amikacin as a core drug were excluded. Outcomes were classified per WHO/PNLT programmatic definitions, and case classification followed routine registry validation. Age and treatment delay categories (3–64 vs 65–75 years; 0–7 vs 8–376 days) were selected a priori based on distributional properties and programmatic cut points.

Variables collected included age, sex, year of diagnosis, residence (urban or rural), health zone, date of sample collection, diagnostic date, diagnostic delay, GeneXpert testing date, treatment initiation date, treatment delay, TB treatment history (new, relapse, failure, return after loss to follow-up, or other), type of resistance (RR-TB, MDR-TB, pre-XDR-TB), HIV status (positive, negative, or unknown), and treatment regimen received (including bedaquilline + clofazine + moxifloxacine/levofloxacine + ethambutol + pyrazinamid + isoniazid (HD) + prothionamid/ethionamid; BPaL, or BPaLM) [13]. Treatment outcomes were classified according to WHO definitions as successful (cured and/or treatment completed) or unsuccessful (death, treatment failure, LTFU, or treatment refusal). Cohort exit date and treatment duration were also recorded. For analytical purposes, patients were categorized into incident cases (new and relapse) versus retreatment cases excluding relapse (failure, return after loss to follow-up, or other).

Categorical variables were summarized as proportions, while continuous variables were expressed as medians with interquartile ranges (IQRs). Associations were tested using the χ² or Fisher's exact test for categorical variables and the Wilcoxon rank-sum test for continuous variables. Logistic regression was used to estimate unadjusted associations between baseline variables and treatment outcomes. Multivariable logistic regression models were then fitted, including all baseline covariates a priori. Backward elimination was applied, and the final model retained statistically significant predictors along with variables considered clinically relevant based on prior evidence [2, 3, 5].

Patients with unsuccessful outcomes (death, treatment failure, LTFU, or treatment refusal) were analyzed as a composite category due to small subgroup sizes. Time-to-event analyses were performed using Kaplan–Meier estimates, with survival distributions compared across groups using the log-rank test. Univariable and multivariable Cox proportional hazards models were used to identify independent predictors of unsuccessful outcomes, with results expressed as adjusted hazard ratios (aHRs) and 95% confidence intervals (CIs). Analyses were based on complete cases; no imputation was performed. Multicollinearity was assessed using variance inflation factors (all VIFs < 2). For Cox models, proportional hazards assumptions were tested using Schoenfeld residuals with no major violations detected. In sensitivity analyses, we fit models with standard errors clustered by health zone; inferences were unchanged (Supplementary Figure 1). A 2-sided *P* value < .05 was considered statistically significant. All analyses were conducted using R version 4.3.3 (R Core Team, 2023)

## RESULTS

Of 314 DR-TB notifications identified, 37 were excluded (30 were treated with injectable drug, amikacin as a core drug, 3 duplicates, 4 missing critical outcome dates), leaving 277 DR-TB for analysis, with a male-to-female ratio of 2.9:1.

The overall treatment success rate was 77.6%, while 13.4% of patients died (26% among them before treatment initiation) and 7.9% were LTFU and 1.1 were failure and treatment refused. The median age of the cohort was 36 years (range: 3–75 years). Among participants, 11.6% had an unknown HIV status, and DR-TB/HIV co-infection was identified in 9.4% of the cohort, all of whom initiated antiretroviral therapy (ART). Treatment success was achieved in 19 (73.1%) of the 26 co-infected patients, with 5 (19.2%) deaths and 2 (7.7%) LTFU. The mortality rate in this co-infected group was higher than in the general DR-TB cohort (13.4%).

The mean turnaround time for molecular test results using GeneXpert was 4 days (range: 0–124 days). Total follow-up amounted to 7.77 person-months (median treatment duration 275 days, IQR 274–278 among successes; 33 days, IQR 13–127 among unsuccessful). The median interval from diagnosis to treatment initiation was 7 days (IQR: 3–17), although delays of up to 367 days were observed. When age was categorized into 2 groups (3–64 and 65–75 years), older patients grouped in the over-64 age category, considered stable, were more likely to experience unfavorable outcomes in urban, although due to the small sample size in the latter group (n = 8), differences between the cohorts were borderline statistically significant (*P* = .059). Bedaquiline was prescribed as a core drug in 98.9% of cases, while 2 patients with pre-XDR-TB were treated with the BPaLM regimen and 1 with BPaL.

In Table 1, the comparison of favorable (cured or treatment completed) versus unfavorable outcomes (death, LTFU, treatment failure, or no treatment) also demonstrated significant associations with place of residence (*P* = .001), treatment duration (*P* < .001), age (*P* = .046), and treatment history (*P* = .026).

**Table 1.** Characteristics of Drug-Resistant TB Patients in South Kivu by Treatment Outcomes (2018–2024)

Characteristic	Successful (n = 215)^a^	Unsuccessful (n = 62)^a^	*P* Value^b^
Residence			.001
City	37 (17.2%)	20 (32.3%)	
Rural	178 (82.8%)	42 (67.7%)	
Sex			.40
Female	57 (26.5%)	20 (32.3%)	
Male	158 (73.5%)	42 (67.7%)	
Age, median (IQR) year	37 (30–45)	32 (27–44)	.046
Age group			.70
3–64 y	208 (96.7%)	61 (98.4%)	
65–75 y	7 (3.3%)	1 (1.6%)	
HIV status			.80
Negative	170 (79.1%)	49 (79.0%)	
Positive	19 (8.8%)	7 (11.3%)	
Unknown	26 (12.1%)	6 (9.7%)	
Treatment delay, median (IQR), days	7 (3–17)	6 (3–12)	.60
Treatment duration, median (IQR), days	275 (274–279)	33 (13–127)	<.001
Treatment duration group, days			.40
0–7 d	113 (52.6%)	36 (58.1%)	
8–376 d	102 (47.4%)	26 (41.9%)	
Resistance profile (Xpert MTB/RIF, Xpert MTB/Ultra, and Xpert MTB/XDR)			.60
RR	212 (98.6%)	61 (98.4%)	
INH mono-resistance	2 (0.9%)	1 (1.6%)	
Pre-XDR	1 (0.5%)	0 (0.0%)	
Core regimen drug			.30
BDQ	213 (99.1%)	61 (98.4%)	
BPaL	0 (0.0%)	1 (1.6%)	
BPaLM	2 (0.9%)	0 (0.0%)	
Diagnostic delay, days			>.9
0–2 d	184 (85.5%)	53 (85.5%)	
3–124 d	31 (14.5%)	9 (14.5%)	
TB treatment history			.029
New/relapse	156 (72.6%)	36 (58.1%)	
Retreatment (other)	59 (27.4%)	26 (41.9%)	

Genotype: RR-TB, rifampicin-resistant; pre-XDR-TB per WHO 2020 + definitions. Regimens: BDQ; BPaL/BPaLM as defined. Missing: HIV n = 32.

^a^n (%); median (Q1, Q3).

^b^Fisher's exact test; Pearson's χ² test; Wilcoxon rank-sum test.

In the adjusted analysis (Table 2), rural residence was associated with higher odds of treatment success compared to urban areas (aOR = 0.42; 95% CI: 0.21–0.82; *P* < .011). Retreatment cases excluding relapse had significantly higher odds of unsuccessful outcomes (aOR = 2.04; 95% CI: 1.09–3.81; *P* = .026). HIV status and age were included in the multivariable model but were not significantly associated with outcome.

**Table 2.** Logistic Regression of Factors Associated With Unsuccessful Outcomes Among DR-TB Patients in South Kivu (2018–2024)

Variable	Crude OR (95% CI)	*P*	Adjusted OR (95% CI)	*P*
Sex				
Female	1.00 (ref)	…	…	…
Male	…	…	0.76 (0.41–1.42)	.37
Age	0.98 (0.96–1.00)	.11	0.98 (0.96–1.01)	.2
Age group	…	…	…	…
3–64 y	1.00 (ref)	…	…	…
65–75 y	0.49 (0.03–2.81)	.51	…	…
Treatment delay group (days)				
0–7	1.00 (ref)	…	…	…
8–376	0.80 (0.45–1.41)	.44	…	…
Diagnostic delay group (days)				
0–2	1.00 (ref)	…	…	…
3–124	1.01 (0.43–2.17)	.98	…	…
HIV				
Negative	1.00 (ref)	…	1.00 (ref)	…
Positive	1.28 (0.48–3.09)	.60	1.02 (0.36–2.62)	>.9
Unknown	0.80 (0.29–1.94)	.64	0.54 (0.18–1.44)	.20
Residence				
Urban	1.00 (ref)	…	1.00 (ref)	…
Rural	0.44 (0.23–0.84)	.01	0.42 (0.21–0.82)	.011
Treatment history				
News/relapse	1.00 (ref)	…	1.00 (ref)	…
Retreatment	1.91 (1.06–3.43)	.031	2.04 (1.09–3.81)	.026

Logistic regression reporting OR and aOR (95% CI). The multivariable model adjusted a priori for sex, age group, residence (urban/rural), HIV status, diagnostic delay, treatment delay, and TB treatment history (incident vs retreatment excluding relapse). Successful versus unsuccessful outcomes as defined in Table 1.

Cox regression analysis (Table 3) confirmed these findings. Living in a rural health zone was independently associated with faster achievement of treatment success compared to urban residence (aHR = 0.50; 95% CI: 0.29–0.85; *P* = .011). Being a new or relapse case was also associated with more favorable outcomes compared with retreatment cases other than relapse (aHR = 1.71; 95% CI: 1.03–2.83; *P* = .038).

**Table 3.** Cox Regression Analysis of Predictors of Unfavorable Outcomes Among DR-TB Patients in South Kivu (2018–2024)

Variable	Crude HR (95% CI)	*P*	Adjusted HR (95% CI)	*P*
Sex				
Female	1 (ref)	…	…	…
Male	0.71 (0.41–1.22)	.2	…	…
Age group				
3–64 y	1 (ref)	…	…	…
64–75 y	0.66 (0.07–5.83)	.7	…	…
Treatment delay group (days)				
0–7 d	1 (ref)	…	…	…
8–376 d	1.00 (0.57–1.75)	>.9	…	…
Diagnostic delay group (days)				
0–2 d	1 (ref)	…	…	…
3–124 d	1.19 (0.57–2.48)	.7	…	…
HIV				
Negative	1 (ref)	…	1 (ref)	…
Positive	0.98 (0.42–2.25)	>.9	0.59 (0.26–1.36)	.22
Unknown	0.65 (0.27–1.60)	.4	0.77 (0.37–1.62)	.50
Residence				
Urban	1 (ref)	…	1 (ref)	…
Rural	0.48 (0.27–0.83)	.008	0.50 (0.29–0.85)	.011
Treatment history				
News/relapse	1 (ref)	…	…	…
Retreatment	1.94 (1.13–3.33)	.016	1.71 (1.03–2.83)	.038

Cox proportional hazards models reporting HR and aHR (95% CI) for time to unsuccessful outcome (death, failure, LTFU, not evaluated). Adjusted for sex, age group, residence, HIV status, treatment delay, and TB treatment history. Proportional hazards assumptions verified with Schoenfeld residuals; sensitivity analyses with SEs clustered by health zone yielded unchanged inferences.

Time-to-event analyses (Table 3; Supplementary Figure 1) were concordant. Rural residence remained protective (aHR 0.50; 95% CI 0.29–0.85; *P* = .011), and retreatment excluding relapse increased risk (aHR 1.71; 95% CI 1.03–2.83; *P* = .038). Kaplan–Meier curves demonstrated clear separations for treatment category (Supplementary Figure 1*A*, log-rank *P* = .002) and residence (Supplementary Figure 1*B*, *P* = .04); HIV status (Supplementary Figure 1*C*) showed no significant separation probably due to missing data n = 37 (11.8%); and final outcome (Supplementary Figure 1*D*) showed expected divergence (*P* < .001). All other baseline variables < 5% missing. Findings were robust in sensitivity analyses excluding pretreatment deaths and when clustering by health zone.

## DISCUSSION

This province-wide analysis (2018–2024) shows a treatment success rate of 77.6%, in line with the WHO ≥ 75% benchmark for MDR/RR-TB, but with mortality of 13.4% and LTFU of 7.9%, underscoring persistent gaps in the care cascade [1]. The improvement relative to the 2012–2017 South Kivu cohort (≈70.8% success) likely reflects the transition from long, injectable-based regimens to shorter, fully oral regimens that are better tolerated and easier to deliver programmatically [5, 7, 14, 15]. Bedaquiline was introduced for the treatment of MDR-TB in South Kivu in 2016, subject to specific eligibility criteria (recommended exclusively for new RR-TB cases). Following the scaling up of its use in 2018, the province transitioned to all-oral regimens, culminating in the complete phasing out of injectable drug by 2023. This transition was implemented gradually from 2018, depending on the patient type and treatment center (hospital or health facility center). Nevertheless, deaths occurring before treatment initiation in our cohort indicate ongoing bottlenecks in timely diagnosis and drug availability amid health-system disruptions (COVID-19, mpox) and insecurity due to armed conflicts in the South Kivu Province [4, 8, 9].

Two independent predictors of unfavorable outcomes were identified: urban residence and retreatment other than relapse. An inequitable distribution of Xpert machines, which are more readily available in urban areas compared to rural areas and the cyclical nature of conflict in Kivu, coupled with the systemic destruction of rural healthcare infrastructure, has forced populations to seek medical care in urban centers. However, the extended duration of TB treatment, paired with the loss of livelihoods, exposes patients to severe economic precarity in cities, compelling them to return to their villages and subsequently leading to high TB treatment default rates.

The urban disadvantage contrasts with the often-presumed access advantages of cities and may be explained by the high mobility of patients where a predominantly male demographic (2.9:1 ratio, miners, displaced persons) who are diagnosed in urban centers but interrupt care when returning to rural homes or mining. This lack of formal referral mechanisms or continuity mechanisms not only hinders clinical completion but also facilitates ongoing disease transmission within these highly mobile, high-risk social networks. Similar links between mobility/urban settings and poorer outcomes have been documented in Estonia and across several African settings [16–19]. Internally displaced persons (IDPs) in Syrian villages and cities showed a decreased risk of poor treatment outcomes (30% and 13% lower, respectively) compared to camp residents [20], In contrast, IDPs in Pakistan experienced elevated rates of treatment failure and default [21].

The strong association of prior TB treatment with failure, non-adherence, or mortality in our data aligns with evidence from China, Ghana, Ethiopia, and Uganda, where prior treatment history consistently predicts worse DR-TB outcomes [19, 22, 23] and non-adherence [24]. Also, our results are consistent with Cameroon database trends showing persistent risks among retreatment cases and mobile populations and with Sudan causal analyses indicating prior TB treatment increases the probability of adverse DR-TB outcomes [25, 26].

Our outcomes are broadly comparable to those reported in Tanzania and Ethiopia (success ≈75%–77%; mortality ≈9%–10%) [14, 15] and exceed success rates observed in Indonesia, India, Pakistan, and China (≈50%–65%) [27–30]. At the same time, they fall short of the highest programmatic performances reported in Ethiopia and Pakistan (≥83%–90%) and pooled meta-analytic estimates (≈83%) [5, 31]. Taken together, these comparisons suggest that scale-up of short, all-oral regimens, coupled with community-based support, can achieve robust effectiveness in fragile settings, but continuity of care for mobile and retreatment populations remains a critical obstacle [5, 7, 32].

Delays and early events merit attention. Although median time from diagnosis to treatment was short overall (7 days), the wide tail of delays and the subset of pretreatment deaths indicate missed opportunities for accelerated linkage to care and reliable drug supply. This pattern mirrors prior reports that early mortality and early LTFU in DR-TB are often related to late presentation and system bottlenecks, particularly in resource-constrained or crisis-affected contexts [6, 8, 33]. Strengthening referral feedback loops, patient tracing, and buffer stocks for key drugs may reduce these preventable losses.

This study has several notable strengths. First, it represents one of the largest province-wide DR-TB cohorts reported from the DRC, encompassing around 300 patients across multiple health zones. Second, it directly compares outcomes with a previous provincial cohort (2012–2017) [8], thus documenting progress over a decade. Third, the analysis incorporated both logistic regression and Cox proportional hazards models, which consistently identified the same predictors, increasing robustness of findings. Finally, the study captures real-world programmatic conditions in a fragile, conflict-affected setting, making its results highly relevant to other humanitarian contexts where evidence is scarce.

Although informative, our study is limited by its retrospective design and reliance on routine registry data, which precluded analysis of potentially important variables (eg, adverse drug reactions, nutrition, socioeconomic status, occupation/mining exposure) [4, 8]. Approximately 10% with unknown HIV status could introduce misclassification, although HIV was not an independent predictor in adjusted models. Referral and recording practices may underestimate LTFU among highly mobile patients who leave without notifying providers. Finally, as a single-province analysis, generalizability to other DRC provinces should be cautious, though findings likely apply to comparable conflict-affected, high-burden settings [1, 9].

Clinical and public health policy implications: the findings carry important implications for clinical practice and public health policy. The high overall success rate supports the scale-up of short, fully oral regimens (BPaL/BPaLM) across DRC and similar settings. However, the disproportionate burden of poor outcomes in urban residents and retreatment cases highlights the need for targeted interventions, including enhanced patient-tracing systems, referral feedback mechanisms, and adherence support tailored to mobile and mining populations. Clinically, early mortality underscores the urgency of reducing diagnostic-to-treatment delays through rapid GeneXpert scale-up, same-day treatment initiation, and uninterrupted drug supply chains. At the policy level, sustained donor investment and integration of TB programs within universal health coverage frameworks are essential to protect recent gains and advance toward End TB milestones.

## CONCLUSION

This study demonstrates that while a short, all-oral treatment regimen for DR-TB in South Kivu achieved therapeutic success rates exceeding WHO targets, significant challenges remain compared to drug-susceptible TB. High rates of mortality and loss-to-follow-up persist, primarily driven by retreatment history and suboptimal urban referral systems. The inherent complexity of RR/MDR-TB management requiring rapid molecular diagnostics and rigorous clinical monitoring remains highly susceptible to the disruptions of conflict, population displacement, and fragile health infrastructure. These findings underscore the urgent need for context-adapted strategies and further development of ultra-short, all-oral regimens to enhance treatment adherence and advance global TB elimination goals in humanitarian settings.

## Supplementary Material

ofag303_Supplementary_Data

## Supplementary Data

Supplementary materials are available at *Open Forum Infectious Diseases* online. Consisting of data provided by the authors to benefit the reader, the posted materials are not copyedited and are the sole responsibility of the authors, so questions or comments should be addressed to the corresponding author.
