# Supplementary figures and images for: Drug-Resistant Tuberculosis Outcomes in South Kivu Province, Democratic Republic of Congo, During a Humanitarian Crisis (2018–2024)
Source: Open Forum Infect Dis. 2026 May 14;13(5):ofag303. doi: 10.1093/ofid/ofag303 (PMC13221644; doi:10.1093/ofid/ofag303)

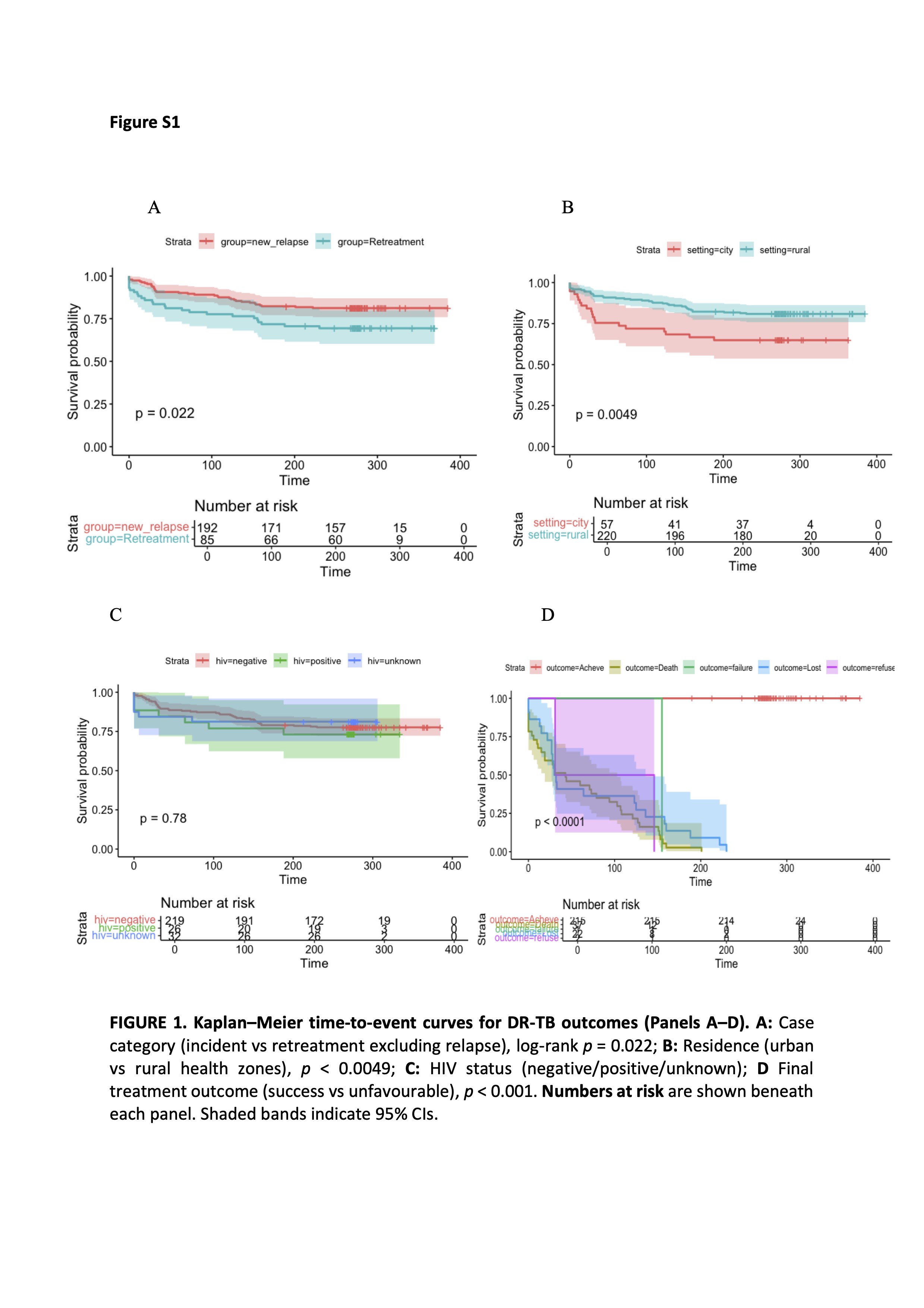

Supplement: ofag303_Supplementary_Data [file ofag303_supplementary_data.jpeg]
